# The Impact of the Laterality on Radiographic Outcomes of the Bernese Periacetabular Osteotomy

**DOI:** 10.3390/jpm12071072

**Published:** 2022-06-29

**Authors:** Carsten Y. W. Heimer, Chia H. Wu, Carsten Perka, Sebastian Hardt, Friedemann Göhler, Henrik C. Bäcker

**Affiliations:** 1 Department of Orthopaedic Surgery and Traumatology, Charité Berlin University Hospital, 10117 Berlin, Germany; carsten.perka@charite.de (C.P.); sebastian.hardt@charite.de (S.H.); henrik.baecker@sports-med.org (H.C.B.); 2 Department of Orthopaedics & Sports Medicine, Baylor College of Medicine Medical Centre, Houston, TX 77030, USA; wu.chia.h@gmail.com; 3 Department of Radiology, Charité Berlin University Hospital, 10117 Berlin, Germany; friedemann.goehler@charite.de

**Keywords:** radiography, periacetabular osteotomy, PAO, dysplasia, complication, laterality

## Abstract

The purpose of this study was to compare the pre and postoperative radiographic findings and analyze the complication rate with respect to the laterality in periacetabular osteotomy in right-handed surgeons. Satisfaction rate and radiographic findings were prospectively collected between 2017 and 2019 and retrospectively reviewed. For analysis, all measurements of the CT scans were performed by a musculoskeletal fellowship-trained radiologist. Complications were classified into two categories: perioperative or postoperative. All surgeries were performed by three right-hand dominant hip surgeons. A total of 41 dysplastic hips (25 right and 16 left hips) in 33 patients were included. Postoperatively, a significantly lower acetabular index angle on the left side was observed at −2.6 ± 4.3 as compared to the right side at 1.6 ± 6.5 (*p* < 0.05). The change in Center edge (CE) angle was significantly lower for the left side 13.7 ± 5.5° than on the right side, measured at 18.4 ± 7.3 (*p* < 0.001); however, the overall CE angle was comparable at 38.5 ± 8.9° without any significant difference between the operated hips (left side at 37.8 ± 6.1° versus right side at 39.0 ± 10.3; *p* = 0.340). No significant differences in other radiographic measurements or surgical time were observed. For complications, the right side was more commonly affected, which may also explain a higher satisfaction rate in patients who were operated on the left hip with 92.3%. The change in lateral CE angle was significantly lower for the left side and the right hip seems to be predisposed to complications, which correlate with a lower satisfaction rate in right-handed surgeons.

## 1. Introduction

One of the most common causes of secondary osteoarthritis is developmental dysplasia of the hip [1]. To manage the risk of early-onset osteoarthritis, periacetabular osteotomy (PAO) is the method of choice. Initially described by Ganz in the 1980s, the Bernese PAO allows for repositioning the acetabulum to reduce super-lateral acetabular inclination and improve femoral head coverage, thus restoring the joint center [2]. Indications consist of persistent pain, age between 15 and 45 years of age, positive impingement test, good range of motion, lack of severe osteoarthritis (Kellgren–Lawrence score less than 2), crossing-over sign, and presence of center edge (CE) angle less or equal to 25° [3]. 

Surgeons try to achieve a horizontal acetabular weight-bearing area [4] with PAO while ensuring that the osteotomy sites stay in contact to avoid nonunion. The acetabular inclination (AI) angle should be between 0° and 10°, whereas the CE angle is aimed to be approximately 35° for the best outcome, according to Hartig-Andreasen [5].

The current literature supports the use of PAO for correcting radiographic deformity and improvement in hip function can be inferred by a low total hip arthroplasty (THA) conversion rate between 0 and 17% of cases [6,7,8]; however, the complication rate can be as high as 37% according to some studies, in part due to the steep learning curve [9,10]. To improve satisfaction, factors other than radiographic angles and indices, such as the presence of an aspherical femoral head, have to be considered [6].

When looking for total hip arthroplasties, an impact of the laterality, as well as surgeon’s handedness, was observed, especially in the positioning of the cup [11]. Hereby, an inclination angle of the acetabular component revealed 46.4° on the dominant operated side compared to 43.5° to the non-dominant side [12].

For periacetabular osteotomy, an accurate reorientation of the acetabulum is even more important to avoid osteoarthritis. To our knowledge, no study to date has investigated the laterality as one of the factors in determining the radiographical outcomes.

For PAO, this study analyzes the laterality of surgery on (1) the radiographic outcome, including the positioning of the socket and (2) complication rate.

## 2. Materials and Methods

A retrospective study was conducted to evaluate the outcomes of consecutive patients undergoing the Bernese periacetabular osteotomy between 2017 and 2019. Inclusion criteria consisted of complete pre and postoperative assessment of patients older than 18 years of age with a minimum follow-up of 6 months. Patients younger than 18 years of age, with incomplete records (such as missing anteroposterior radiography of the hip instead of the pelvis), or with shorter follow-up were excluded.

To undergo surgery, patients have to meet hip dysplasia criteria published by Tannast et al., have no signs of severe radiographic osteoarthritis, and endured consistent hip pain for at least 1 year [13]. This includes a CE angle of below 20°, an AIA (femoral head bony coverage by the acetabulum) of more than 10°, an AHI (difference between the horizontal line connecting both triradiate cartilages (Hilgenreiner line) and the acetabular roofs) of more than 25% or a sharp angle of more than 42°. Borderline hip dysplasia is defined as a CE angle between 20° and 24.9° and a sharp angle between 39° to 42°, respectively; the presence of a crossing-over sign was used.

All surgeries were performed by one of three experienced right-hand dominant hip surgeons. For preoperative diagnostics, a clinical examination including hip range of motion, quality of pain, body weight (kg), height (cm), body mass index (kg/m^2^), anteroposterior radiography of the pelvis, axial view of the affected hip, and torsional computed tomography were included. Intraoperatively, the surgical time, anesthesia time, as well as the necessity of blood transfusion, was noted. For postoperative radiographs, an anteroposterior view of the pelvis and axial view of the hip were performed.

Radiographic measurements included the center edge angle, acetabular index angle (AIA), sharp angle, hip lateralization, the centrum-collum-diaphyseal angle (CCD) on the anteroposterior view, and the alpha, as well as beta angle on axial X-ray, were obtained. Additionally, the presence of a crossing-over sign and femoral-acetabular impingement (CAM and/or pincer impingement) were noted.

**Figure 1:**
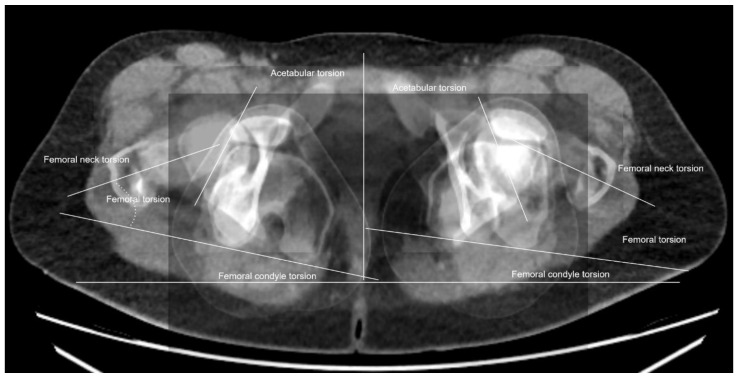
Rotational alignment in a 29-year-old patient with left-sided dysplastic hip: Femoral neck rotation +22°, femoral condyle rotation −24°, femoral rotation +46°, tibial plateau rotation −21°, and femorotibial rotation difference +3°.

**Figure 2:**
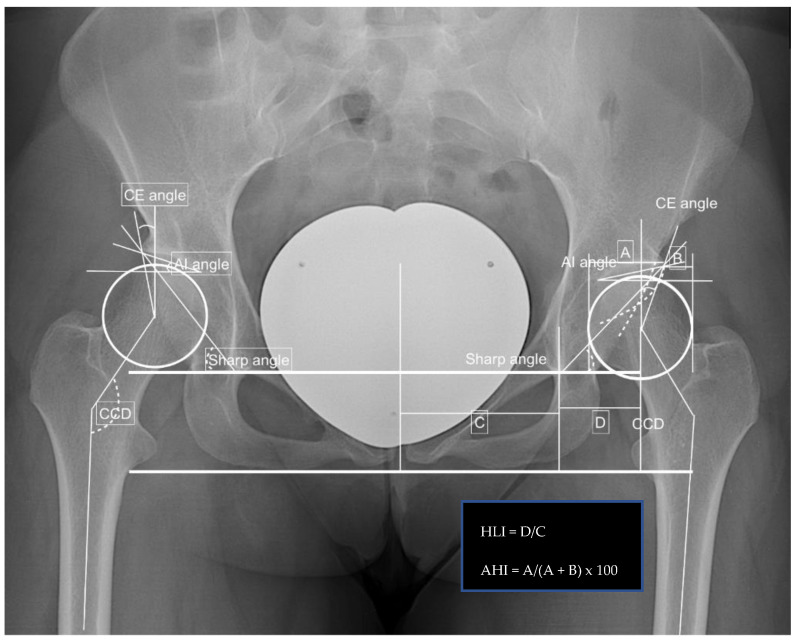
Preoperative findings and measurements performed; left side: CE angle 18.8°, AIA 12.3°, sharp angle 43.9°, hip lateralization 0.54, anterior hip index 73.9, CCD angle 137.4°, and positive crossing-over sign.

**Figure 3:**
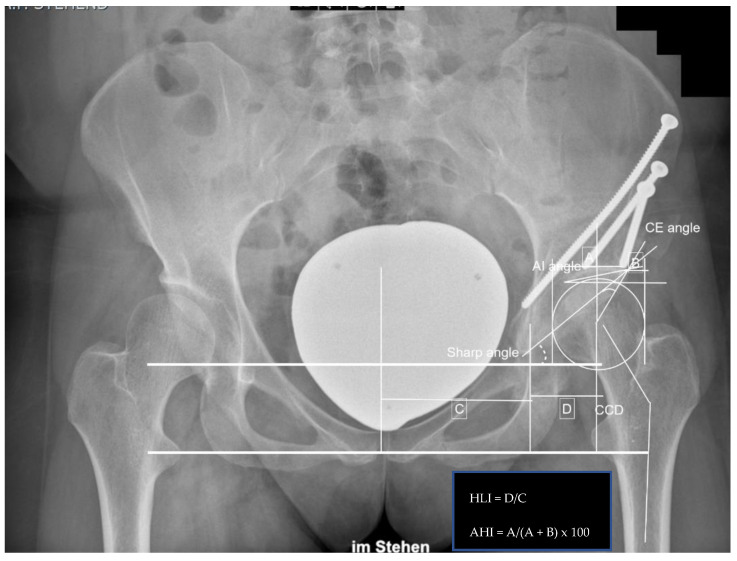
Postoperative measurements after left-sided PAO: CE angle 38.0°, AIA −0.5°, sharp angle 36.5°, hip lateralization 0.56, anterior hip index 91.4, CCD angle 137.4°, and positive crossing-over sign.

**Figure 4:**
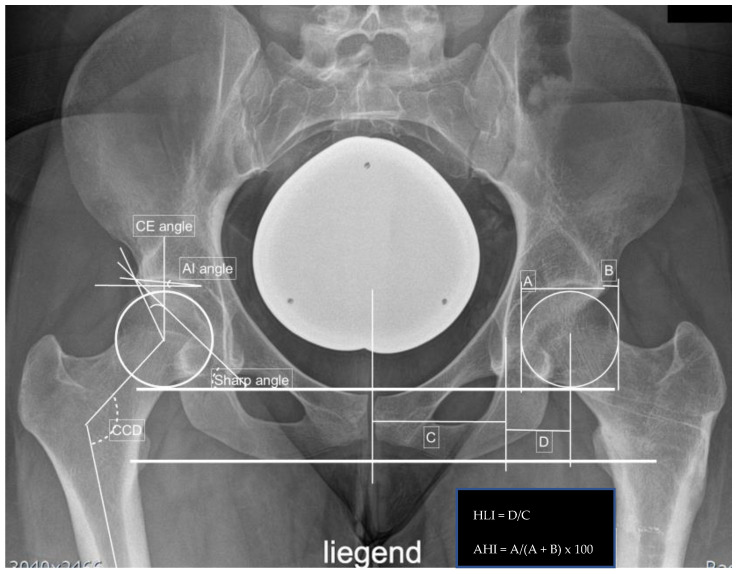
Preoperative measurements in a right-sided borderline dysplastic hip: CE angle 23.4°, AIA 4.9°, sharp angle 40.0°, hip lateralization 0.51, anterior hip index 82.8, and CCD angle 121.0°.

**Figure 5:**
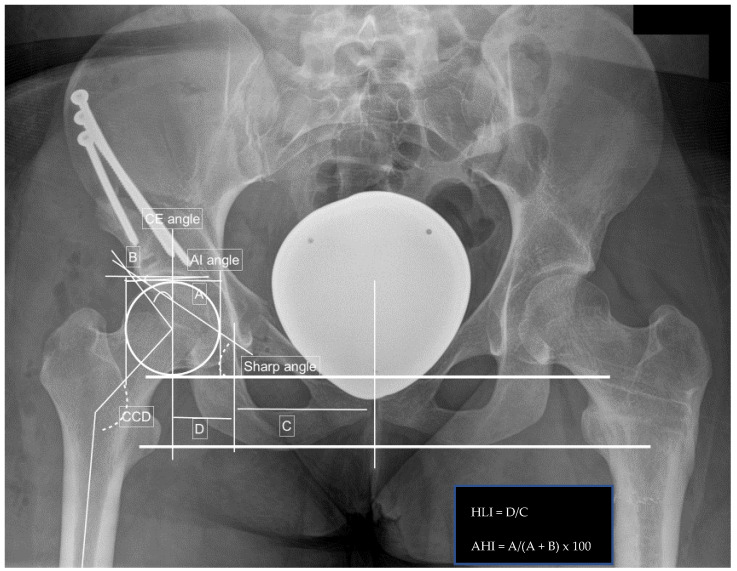
Postoperative measurements after right-sided PAO: CE angle 38.8°, AIA −5.1°, sharp angle 28.0°, hip lateralization 0.57, anterior hip index 90.3, CCD angle 121.0°, and positive crossing-over sign.

For torsional computed tomography, a non-contrast CT of the lower extremity on either a 320-row or an 80-row CT scanner (Canon Aquillon ONE Vision Edition/Canon Aquillon PRIME, Canon Medical Systems, Tochigi, Japan) was performed. The scan was performed with 120 kVp tube voltage and automated tube current modulation set at low dose mode with a 0.5 to 1.0 mm slice thickness using iterative reconstruction and a bone kernel. Measurements were performed by a musculoskeletal fellowship-trained radiologist, including an acetabular rotation at the level of the acetabular center. The angle between the target along the posterior and the anterior acetabular edge and a tangent along the right and left sciatic spine was measured. Likewise, the torsions of the femur, tibia, and tibiofemoral torsion were determined again by an image baseline parallel to the inferior image border. The difference between the femoral neck rotation and the rotation of the femoral condyles resulted in femoral torsion. The tibial torsion was obtained as the differences between tibial plateau rotation and the rotation of the upper ankle. Finally, the tibiofemoral rotation was calculated as the difference between the femoral condyle rotation and the tibial plateau rotation. All rotational measurements are illustrated in Figure 1. In addition, Figure 2, Figure 3, Figure 4 and Figure 5 show two examples of left, respectively, right-sided dysplastic/borderline dysplastic hip before and after surgery.

During follow-up, the functional outcome was classified into (1) very satisfied, (2) satisfied, (3) neither satisfied nor dissatisfied, and (4) dissatisfied. The complications were defined as either perioperative, such as bleeding that required blood transfusion, or postoperative complications, such as wound healing issues and postoperative hematoma. In addition, the hardware removal rate related to discomfort was recorded.

For statistical analysis, Microsoft Excel (Version 16.36, Microsoft Corporation, Redmond, WA 98052-6399, USA), IBM^®^ SPSS^®^ Statistics 26 Core System (IBM, Armonk, NY, USA), and Origin Pro 8.0 (OriginLab Cooperation, Northampton, MA 01060, USA) were used. The mean and standard deviation of the mean (SD) are presented for all normally distributed continuous variables. A multivariate Analysis of Variance (ANOVA) T-Test was applied, and level of significances were set to * *p* < 0.05, ** *p* < 0.01, and *** *p* < 0.005. All values are recorded to one decimal place. An a priori power analysis was shown when using conservative estimation to achieve 80% power at a 5% significance level; an effect size of 1.5 can be assumed, requiring 8 subjects (PASS 2008).

## 3. Results

Between 2017 and 2019, a total of 41 hips in 33 patients were included. Of the 41 hips, 25 right and 16 left hips underwent PAO. The mean age was 28.6 ± 7.4 years without any significant differences between the two groups. The mean age of the left hip group was 29.4 ± 7.7 as compared to the right side at the age of 28.1 ± 7.1 (*p* = 0.288). Females gender consisted of 90.1% of cases with no difference among the two groups (left side 100.0%, right side 80.8%; *p* = 0.048).

Similarly, no differences were observed for height at 169.7 ± 8.3 cm (left side 168.1 ± 7.6 cm, right side 170.7 ± 8.5 cm; *p* = 0.178), body weight at 68.0 ± 11.7 kg (left side 65.6 ± 9.3 kg, right side 69.6 ± 12.7 kg; *p* = 0.159), and body mass index at 23.6 ± 3.8 kg/m^2^ (left side 23.5 ± 3.1 kg/m^2^, right side 69.6 ± 12.7 kg/m^2^; *p* = 0.450). In our cohort, borderline dysplastic hips were diagnosed in 68.3% of patients (left hip group with 75.0%, *n* = 12/16; right hip group with 64.0% *n* = 16/25; *p* = 0.236). Depression was observed in seven cases (17.1%, *n* = 7/41), which were equally distributed between the two groups: left side at 18.8% (*n* = 3/16) and right side at 16.0% (*n* = 4/25). Other comorbidities included asthma bronchiale in two cases (*n* = 2/41, 4.9%, one in each group). There is one Crohn’s disease patient in the left hip group (*n* = 1/16) and one case of mitral regurgitation in the right hip group (*n* = 1/25). For fixation of the socket, screws were used in 85.4% (*n* = 35/41, left hip group 87.5%, *n* = 14/16, respectively, right hip group 84.0%, *n* = 21/25) of cases. In the remaining cases k-wires were used for fixation. All demographics are illustrated in Table 1.

For follow-up, the mean was 267.5 ± 208.5 days with no significant difference between the two groups (left side 222.7 ± 251.0 days versus 296.7 ± 169.0 days; *p* = 0.149). In all patients, the union was observed at their final follow-up.

Preoperatively, significant differences between the two groups were observed for CE angle and alpha angle on radiography. The CE angle was 22.0 ± 6.2° overall (left group 24.1 ± 5.2° versus right group 20.7 ± 6.5°; *p* = 0.045). The alpha angle was 100.7 ± 10.7 overall (left group 97.0 ± 9.5° versus right group 103.4 ± 10.7; *p* = 0.034). The rotational alignment revealed no significant differences between the groups preoperatively. The findings are summarized in Table 2.

After surgery, a significant difference was observed for the acetabular index angle (AIA) with a mean of −0.02 ± 6.1 and a *p*-value of 0.016 (left side −2.6 ± 4.3 compared to 1.6 ± 6.5 on the right side).

When comparing the radiographic changes before to after the periacetabular osteotomy, significant differences were noted for all parameters measured except the beta angle, which has a *p*-value of 0.210. Likewise, similar findings were observed for the individual groups. The left side changed significantly in all radiographic measurements except the CCD, alpha, and beta angle with *p*-values of 0.179, 0.241, and 0.163, respectively. For the right side, no significant differences were observed for the CCD and beta angle with *p*-values of 0.081 and 0.411, respectively (see Table 3).

Although significant differences were observed in all cases, the positioning of the left side was more accurate to the estimated CE-angle of approximately 35°. All measurements are illustrated in Table 3 and changes in radiographic parameters are summarized in Table 4.

For fixation of the acetabular fragment, screws were used in 85.4% of cases (left side *n* = 14/16, 87.5% versus right side *n* = 21/25, 84.0%, *p* = 0.382). The mean surgical time was 89 ± 32 min with no significant differences (left group 85 ± 22 min versus 91 ± 37 min, *p* = 0.290). The mean anesthesia time was 148 ± 41 min (left side 143 ± 27 min compared to 152 ± 47 min, *p* = 0.260).

Due to intraoperative bleeding, transfusion was required in seven cases (*n* = 7/41, 17.1%). Of this cohort, the right hip was involved in six cases (*n* = 6/25, 24.0% compared to *n* = 1/16, 6.3%, *p* = 0.07). Other complications included hypesthesia of the lateral cutaneous femoral nerve in 37 cases (*n* = 37/41, 90.2%, left side *n* = 14/16, 87.5% versus right side *n* = 22/25, 88.0%, *p* = 0.482); one common peroneal nerve palsy and one postoperative infection that required debridement (*n* = 1/25, 4.0%). Furthermore, two cases of loss of implant fixation were observed, with one in each group (4.9%, left side *n* = 1/16, 6.3% compared to the right side *n* = 1/25, 4.0%, *p* = 0.376). Hardware removal was performed in nine right hips and one left hip due to irritation. No significances were observed (*p* = 0.161) between groups for satisfaction score, with a total of 39.0% of patients who were very satisfied, 22% reporting satisfied, 7.3% reporting neither satisfied nor dissatisfied, and 17.1% reporting dissatisfied. Patients who underwent right-sided PAO were dissatisfied at a rate of 24.0% (*n* = 6/25) as compared to 6.3% (*n* = 1/16) (*p* = 0.25). All complications are listed in Table 5.

Eight female patients underwent bilateral periacetabular osteotomy by the same surgeon, with significant differences for postoperative AIA and anterior hip index. There was also trending toward significance for all other radiographic measurements. All findings are summarized in Table 6.

## 4. Discussion

This study analyses the impact of the laterality on outcomes following PAO. Our patient cohort suggests that the laterality in PAO has an impact on the degree of correction as well as complication rate. Hereby, the right hip PAO is at higher risk for complications, including intraoperative bleeding (*p* = 0.07) or others (*p* = 0.482); however, without any significance. In addition, the CE angle after positioning of the acetabular fragment is slightly higher compared to the left side, with a significant difference in AIA (*p* = 0.016) and a change in CE angle (*p* = 0.021). In our series, right hip PAO has a higher rate of wound infection, peroneal nerve palsy, and transfusion. This may also explain the lower satisfaction rate in patients who underwent right-sided PAO.

The Bernese periacetabular osteotomy is a well-established method to manage the risk of osteoarthritis. The surgical techniques used are described in Appendix A. Typically, the mean change in acetabular inclination (sharp angle) ranges from 4.5° to 25.9°, leading to a change in lateral CE angle from 20° to 44° and a medial translation of the hip center from 5 mm to 10 mm. This is confirmed by our study with a change in the sharp angle of 12.7 ± 4.0°, whereas our change in CE angle was rather low with a change of 16.6 ± 7.1°. Clinically, pain relief can be observed in short and midterm follow-ups as well as improvement in Harris hip score; however, improvement in hip function was not predictable. Major complications occurred in 6% to 37% of cases, including heterotopic ossifications, wound hematomas, nerve palsies, intraarticular osteotomies, loss of fixation, and malreductions [7,14]. In our cohort, the complication rate, including loss of implant fixation, was 4.9%, infection was 4.0%, peroneal palsy was 4.0%, and the necessity of transfusion was 17.1%. When looking for conversion to hip arthroplasty after PAO, this is described to be 96.1% for 5 years, 91.3% for 10 years, 85.0% for 15 years, and 67.6% for 20 years in one meta-analysis [15].

The conversion rate to total hip arthroplasty ranges from 0% to 17%. [8,14] Although no recommendations for optimal femoral coverage can be found, Hartig-Andreasen described that CE angle improvements less than 30° or more than 40° is a risk factor for conversion to total hip arthroplasty [5]. Overcorrection of the acetabulum may increase the dissatisfaction rate and risk of pincer femoro-acetabular impingement (FAI) syndrome as well as osteoarthritis. It is also possible to develop pincer FAI syndrome in postoperative CE angle over 46° [16]. Other radiographic measurements described by Tannast et al. for over-covered hips included a lateral center edge angle between 34° and 39° and an acetabular index between −7° to 2° in non-operated hips. The sharp angle was found to be normal between 38° to 42° and 34° to 37° for over-coverage [13].

For the laterality and surgeon handedness, Moloney et al. were among the first to describe that it may have an impact on surgical outcome [17]. For total hip arthroplasty using the lateral approach, significant differences were observed for the cup inclination angles when a surgeon is operating on the side of the dominant hand; however, the differences may not be clinically significant [12].

This study is limited by small sample size and lack of a case match control arm. In addition to the inclination angle, surgeons are more likely to use a larger ante version on the non-dominant hip (*p* = 0.043). Furthermore, the authors reported a more accurate result when operating on their dominant side [11]. The interplay between surgeon handedness and laterality of the hip undergoing surgery can result in lower abduction and less combined Lewinnek outliers [18].

A possible explanation for the differences in laterality may result from the technique used for reorientation of the osteotomized acetabular fragment, such as a supra-acetabular Schanz screw, until optimal femoral coverage is obtained. Alternatively, a laminar spreader can be used to re-orientate the acetabular fragment, which allows finer adjustment. Once the most suitable position is found, fixation is performed using either a combination of K-wires or screws. Since the surgeon aims for an increased CE angle by pushing the fragment downwards, this may explain the higher CE angle for the dominant right hip for right-handed surgeons. Other factors that may influence the final position are the necessity for bony contact to allow for healing as well as soft tissue constraints. To assess the correction of the acetabulum more accurately, an intraoperative anteroposterior pelvic radiograph could be obtained instead of fluoroscopy. This allows comparing the preoperative imaging with the intraoperative findings better.

In our cohort, no significant differences in demographics between the two groups were found. Preoperatively, CE angles were significantly higher in the left hips compared to the right one (*p* = 0.045). This may result from more right leg dominant patients correlated with more severe symptoms. Postoperatively, higher CE angles were found on the right hip (39.0 ± 10.3°) with significantly higher AIA of 1.6 ± 6.5°. Other trends towards significance were observed for the sharp angle and hip lateralization. When considering Hartig-Andreasen et al.’s recommendations, the left hip seems to have better radiographic parameters postoperatively in right-hand dominant surgeons [5]. In addition, a higher complication rate was observed for the right hip, including more instances of transfusion and a significantly higher rate for peroneal communis nerve palsy. This may also explain the higher numbers of dissatisfied patients in the right hip group.

This study has limitations. Periacetabular osteotomy is a challenging surgery that only a few highly specialized surgeons perform—all of them are right dominant at our center [19,20]. The cohort was rather small and all PAOs were performed by three surgeons; however, the same technique was used since all were trained by the same senior surgeon. In addition, this is a retrospective study with no control arm. Although the complication number was small and the radiographic differences were small, the a priori power analysis showed that at least eight patients are required. A possible explanation for the higher satisfaction in left-sided hips may result from a pivotal reason. Hereby, a higher degree of dysplasia does not necessarily mean a lower CE angle but also a higher acetabular deformity. This could lead to a more challenging periacetabular osteotomy. Lastly, this study also did not look at inter-surgeon variation in technique and fixation method. For the radiographic measurements, it must be mentioned that projection of the radiographs can also affect angulation, therefore impacting our analysis. To minimize the bias of rotation deformity in all patients, a rotational CT was performed. Although we only included patients with a minimum follow-up of 6 months and union was observed in all cases, longer follow-up is needed to investigate the functional outcome.

## 5. Conclusions

In our patient cohort, it appears that right side PAO is at risk for a higher rate of complications. This depends not only on the laterality but also on many factors, including but not limited to surgical technique, fixation method, and patient anatomy. We recommend further studies to evaluate the surgeon handedness on a prospective basis with a matched control arm.

## Figures and Tables

**Table 1:** Demographics of included patients and significance between the two groups.

Observation	Overall	Left Hip	Right Hip	*p*-Value
Numbers (%)	41 (100)	16 (39.0)	25 (61.0)	
Dysplastic hips (%)	13 (31.7)	4 (25.0)	9 (36.0)	0.236
Age (mean age)	28.6 ± 7.4	29.4 ± 7.7	28.1 ± 7.1	0.288
Follow up in days	267.5 ± 208.5	222.7 ± 251.0	296.7 ± 169.0	0.149
Gender (female) *n* (%)	37 (90.1)	16 (100)	21 (80.8)	0.048
Body height (cm)	169.7 ± 8.3	168.1 ± 7.6	170.7 ± 8.5	0.178
Body weight (kg)	68.0 ± 11.7	65.6 ± 9.3	69.6 ± 12.7	0.159
Body mass index (kg/m^2^)	23.6 ± 3.8	23.5 ± 3.1	23.7 ± 4.2	0.450
Mental disorders, Depression *n* (%)	7 (17.1)	3 (18.8)	4 (16.0)	0.412
Fixation (screws) *n* (%)	35 (85.4)	14 (87.5)	21 (84.0)	0.382

**Table 2:** Rotational alignment of the lower extremity before surgery.

Rotational Values	Overall	Left Hip	Right Hip	*p*-Value
Acetabular torsion (°)	18.8 ± 6.9	18.8 ± 6.9	19.3 ± 5.6	0.388
Femoral torsion (°)	30.5 ± 12.9	27.7 ± 14.1	13.3 ± 18.8	0.139
Tibial torsion (°)	37.4 ± 11.0	37.0 ± 7.6	41.8 ± 8.8	0.433
Femorotibial torsion (°)	6.2 ± 5.4	−6.6 ± 11.1	7.8 ± 3.6	0.082

**Table 3:** Radiographic findings pre and postoperatively include significant differences between the individual groups. (Bold values denote statistical significance).

X-ray Measurements	Preoperatively				Postoperatively				Difference
	Overall	Left Hip	Right Hip	*p*-Value	Overall	Left Hip	Right Hip	*p*-Value	*p*-Value
CE angle (°)	22.0 ± 6.2	24.1 ± 5.2	20.7 ± 6.5	**0.045**	38.5 ± 8.9	37.8 ± 6.1	39.0 ± 10.3	0.340	**<0.001**
AIA (°)	11.7 ± 5.7	10.6 ± 4.4	12.4 ± 6.3	0.172	−0.02 ± 6.1	−2.6 ± 4.3	1.6 ± 6.5	**0.016**	**<0.001**
Sharp angle (°)	42.9 ± 3.8	41.7 ± 3.3	43.6 ± 3.9	0.069	30.1 ± 5.0	29.3 ± 5.9	30.7 ± 4.3	0.194	**<0.001**
Hip lateralization	0.56 ± 0.07	0.55 ± 0.06	0.56 ± 0.07	0.363	0.62 ± 0.07	0.60 ± 0.05	0.62 ± 0.09	0.179	**<0.001**
Anterior hip index	75.5 ± 7.5	77.4 ± 6.4	74.3 ± 7.8	0.100	89.7 ± 6.8	90.8 ± 6.2	89.1 ± 7.0	0.229	**<0.001**
CCD angle (°)	134.1 ± 6.0	133.0 ± 4.8	134.8 ± 6.5	0.182	136.5 ± 6.8	134.6 ± 4.9	137.7 ± 7.5	0.083	**0.047**
CAM FAI *n* (%)	17 (41.5)	6 (37.5)	11 (44.0)	0.345					
Pincer FAI	2 (4.9)	0 (0)	2 (8.0)	0.128					
Crossing-over sign	14 (34.1)	5 (31.3)	9 (36.0)	0.381					
Kellgren-Lawrence score 0	25 (61.0)	10 (62.5)	15 (60.0)	0.294					
Kellgren-Lawrence score 1	14 (34.1)	6 (37.5)	8 (32.0)						
Kellgren-Lawrence score 2	2 (4.9)	0 (0)	2 (8.0)						
Alpha angle (°)	100.7 ± 10.7	97.0 ± 9.5	103.4 ± 10.7	**0.034**	90.9 ± 12.9	92.1 ± 17.8	90.4 ± 9.9	0.422	**0.005**
Beta angle (°)	57.5 ± 7.4	56.5 ± 7.0	58.3 ± 7.6	0.234	59.4 ± 5.7	60.4 ± 4.4	59.0 ± 6.2	0.355	0.210

**Table 4:** Changes in radiographic parameters between the two groups. (Bold values denote statistical significance).

X-ray Measurements	Change			
	Overall	Left Hip	Right Hip	*p*-Value
CE angle (°)	16.6 ± 7.1	13.7 ± 5.5	18.4 ± 7.3	**0.021**
AIA (°)	−11.7 ± 5.7	−13.2 ± 6.0	−10.7 ± 5.2	0.093
Sharp angle (°)	−12.7 ± 4.0	−12.5 ± 4.7	−12.9 ± 3.5	0.381
Hip lateralization	0.06 ± 0.06	0.05 ± 0.05	0.06 ± 0.06	0.213
Anterior hip index	14.2 ± 5.2	13.4 ± 5.0	14.8 ± 5.2	0.195
Alpha angle (°)	−3.6 ± 30.3	−0.5 ± 16.5	−4.9 ± 34.24	0.412
Beta angle (°)	4.7 ± 18.5	2.3 ± 6.1	5.7 ± 21.4	0.386

**Table 5:** Complication rate after periacetabular osteotomy. (Bold values denote statistical significance).

Complications	Overall	Left Side	Right Side	*p*-Value
Transfusion (%)	7 (17.1)	1 (6.3)	6 (24.0)	0.07
Hypesthesia of lateral cutaneous femoral nerve (%)	37 (90.2)	14 (87.5)	22 (88.0)	0.482
Implant migration (%)	2 (4.9)	1 (6.3)	1 (4.0)	0.376
Peroneal communis nerve palsy (%)	1 (2.4)	0 (0)	1 (4.0)	**<0.001**
Wound infection (%)	1 (2.4)	0 (0)	1 (4.0)	**<0.001**
Hardware removal (%)	10 (24.4)	1 (6.3)	9 (36.0)	**0.015**

**Table 6:** Findings in patients who underwent bilateral PAO. (Bold values denote statistical significance).

Demographic Data	Overall				Left Hip		Right Hip		*p*-Value
Age (mean age)	29.1 ± 6.9				28.9 ± 6.9		29.3 ± 6.9		0.460
Follow up in days	303.6 ± 243.2				271.4 ± 312.7		335.9 ± 135.7		0.312
Body height (cm)	167.7 ± 6.1				167.8 ± 6.2		167.6 ± 6.1		0.485
Body weight (kg)	67.7 ± 7.2				68.1 ± 7.6		67.3 ± 6.7		0.411
Body mass index (kg/m^2^)	24.1 ± 2.8				24.3 ± 2.9		24.0 ± 2.7		0.432
Comparison	Preoperatively				Postoperatively				Difference
	Overall	Left Hip	Right Hip	*p*-Value	Overall	Left Hip	Right Hip	*p*-Value	*p*-Value
CE angle (°)	20.7 ± 5.8	23.0 ± 5.0	18.4 ± 5.6	0.063	39.2 ± 8.6	38.7 ± 5.0	39.8 ± 11.0	0.413	**<0.001**
AIA (°)	13.6 ± 4.4	11.8 ± 4.0	15.4 ± 3.9	0.056	−0.11 ± 4.8	−2.8 ± 4.0	2.6 ± 4.0	**0.013**	**<0.001**
Sharp angle (°)	43.1 ± 4.2	41.5 ± 3.3	44.7 ± 4.4	0.072	30.1 ± 4.5	28.3 ± 4.0	31.9 ± 4.2	0.060	**<0.001**
Hip lateralization	0.556 ± 0.07	0.56 ± 0.08	0.55 ± 0.06	0.386	0.59 ± 0.05	0.60 ± 0.04	0.59 ± 0.05	0.384	**0.043**
Anterior hip index	73.7 ± 6.4	76.6 ± 5.9	70.8 ± 5.5	**0.039**	90.1 ± 5.7	92.7 ± 4.9	87.5 ± 5.2	**0.037**	**<0.001**
Change in Radiographic Parameters									
CE angle (°)	18.5 ± 6.9				15.7 ± 4.7		21.3 ± 7.5		0.057
AIA (°)	−13.7 ± 5.3				−14.6 ± 5.9		−12.8 ± 4.5		0.268
Sharp angle (°)	−13.0 ± 3.8				−13.2 ± 3.7		−12.8 ± 3.9		0.416
Hip lateralization	0.04 ± 0.06				0.04 ± 0.05		0.04 ± 0.07		0.452
Anterior hip index	16.4 ± 4.2				16.1 ± 4.9		16.7 ± 3.2		0.396
Complications									
Transfusion (%)	2 (12.5)				0 (0)		2 (25.0)		0.074
Hypesthesia of lateral cutaneous femoral nerve (%)	16 (100)				8 (100)		8 (100)		1.000
Implant migration (%)	2 (12.5)				1 (12.5)		1 (12.5)		1.000
Hardware removal (%)	2 (12.5)				0 (0)		2 (25)		0.074
Operative Time									
Surgical time (min)	93 ± 36				89 ± 25		97 ± 44		0.340
Anaesthesia time (min)	152 ± 46				147 ± 31		157 ± 57		0.355

## Data Availability

Not applicable.

## Appendix A

For the periacetabular osteotomy, patients are placed in a supine position on a radiolucent table and the ipsilateral arm is placed across the chest. The operated hip is prepped and draped to visualize the iliac crest and hemipelvis. Hereby, the ipsilateral foot should be included.

A modified Smith–Petersen approach with an incision up to the iliac crest is performed. The fascia over the tensor is incised and the abductor muscles are preserved as well as the lateral femoral cutaneous nerve should be protected proximally around the anterior superior iliac spine. Now the tensor fascial muscle is retracted laterally, and the external oblique muscle is dissected off the iliac crest, which allows accessing the inner pelvis. Distally the rectus abdominal muscle is retracted from the anterior inferior iliac spine following dissection of the iliocapsularis muscle off the anterior hip capsule, allowing the palpation of the calcar femoris. For medial dissection, the quadrilateral surface and pubic root are visualized.

The periacetabular osteotomy is performed with an osteotome beginning with the inferior retro-acetabular osteotomy from the infracotyloid groove towards the middle of the ischial spine. The ischial osteotomy is incomplete, with a depth of about 2.5 cm. After retracting the iliopsoas muscle and femoral neurovascular bundle medially, the ramus pubis is cut. Hereby, the osteotome should exit the medial to the obturator nerve. In a further stage, the iliac osteotomy is performed while preserving the superior gluteal artery and vascular arcade supplying the acetabulum. The medial cortex is cut first, followed by the posterior column at an angle of 120 degrees subchondrally.

To confirm the osteotomy, fluoroscopy is used. Now a Laminar spreader and a Schanz screw (5.0 mm) are placed in the superior aspect of the mobile fragment and used for reorientation; therefore, an inwards turn up to an adequate reorientation by internal rotation, forward tilt/extension, and medial translation. The mobile fragment is fixated temporarily using K-wires. The correction is confirmed with an image intensifier again to evaluate the radiographic measurements following definite fixation with K-wires or several 3.5 mm/4.5 mm fully threaded screws. After irrigation and a final hemostasis, the wound is closed in layers [8,19,20].
